# Heterogeneous Water Diffusion on DWI/ADC in Moderately Differentiated Rectal Adenocarcinoma: A Case Report and Pathologic Correlation

**DOI:** 10.2174/0115734056415771251205095248

**Published:** 2026-03-03

**Authors:** Vu Khac Hoang, Nguyen Van Hung, Lam Viet Anh, Tran Van Nam, Nguyen Dinh Toan, Tran Thi Le, Nguyen Tien Nam, Le Anh Duc

**Affiliations:** 1 Department of Radiology, Functional Exploration and Endoscopy Center, Phenikaa University Hospital, Hanoi, Vietnam; 2 Department of Pathological Anatomy, Phenikaa University Hospital, Hanoi, Vietnam; 3 College of Health Sciences, Vin University, Hanoi, Vietnam

**Keywords:** Diffusion-weighted imaging (DWI), Apparent diffusion coefficient (ADC), Colorectal cancer, Rectal adenocarcinoma, Cell density, Diffusion-restriction

## Abstract

**Background:**

Colorectal tumors are common, and multiparametric Magnetic Resonance Imaging, including Diffusion-Weighted Imaging (DWI), Apparent Diffusion Coefficient (ADC) mapping, and contrast-enhanced sequences can assist in distinguishing benign from malignant lesions. However, certain atypical cases exist in which malignancies do not exhibit restricted water molecule diffusion.

**Case Presentation:**

We present the case of a 66-year-old male with moderately differentiated rectal adenocarcinoma showing heterogeneous diffusion restriction on MRI. The tumor surface demonstrated diffusion restriction (hyperintense on DWI, hypointense on ADC), whereas the base did not. Quantitative ADC analysis showed mean values of 0.82 × 10^-3^ mm^2^/s at the surface and 1.36 × 10^-3^ mm^2^/s at the base.

**Conclusion:**

This case highlights that regions with lower cellularity in moderately differentiated adenocarcinoma may lack diffusion restriction. Correlating imaging findings with histopathological results is essential to prevent diagnostic misinterpretation.

## 1 INTRODUCTION

According to Globocan 2022, colorectal cancer ranks among the most prevalent cancers worldwide, placing within the top three for both incidence and mortality-following lung cancer overall, breast cancer in women, and prostate cancer in men [1]. Histopathologically, colorectal cancer comprises various types, with adenocarcinoma representing the vast majority of all cases [2]. Common clinical manifestations include hematochezia, bowel habit changes, abdominal pain, and unintended weight loss. Diagnosis primarily relies on clinical presentation, endoscopic evaluation, and histopatho-logical confirmation through biopsy. Imaging modalities such as Computed Tomography (CT), Magnetic Resonance Imaging (MRI), and Positron Emission Tomography/CT (PET/CT) assist in localizing lesions, distinguishing benign from malignant pathology, evaluating tumor invasion into adjacent structures, and determining disease stage [3, 4]. Genetic testing may also be indicated when hereditary predisposition is suspected. Multiparametric MRI, including Diffusion-Weighted Imaging (DWI), Apparent Diffusion Coefficient (ADC) mapping, and contrast-enhanced sequences are valuable noninvasive tools for assessing malignancy in general, and colorectal tumors in particular [5]. Malignant tumors typically demonstrate restricted diffusion of water molecules within the extracellular space-appearing hyperintense on DWI, hypointense on ADC maps, and showing contrast enhancement. However, some cases or tumor regions may lack these diffusion-restriction features on MRI, complicating the differentiation between benign and malignant lesions. This report presents a case of rectal cancer exhibiting areas without diffusion restriction on MRI, with imaging findings correlated to histopathological results to elucidate the underlying cause of this observation.

## 2 CASE REPORT

**Fig. (1):**
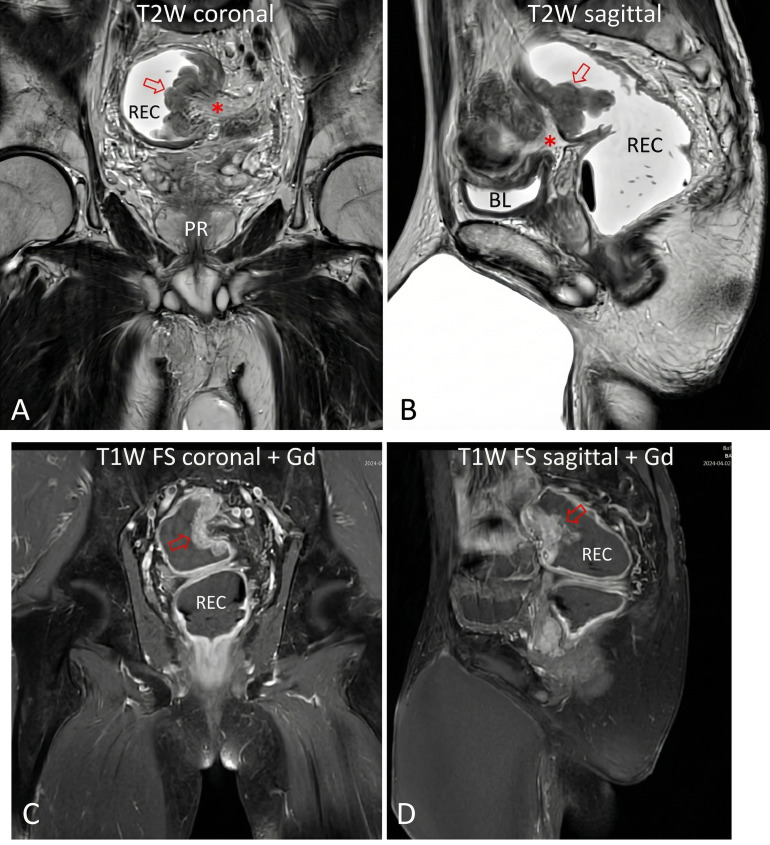
Pelvic MRI with various pulse sequences and planes: (**A**, **B**) T2W coronal and sagittal images demonstrate abnormal thickening of the left anterior wall of the middle third of the rectum over a 6-cm segment, forming an intraluminal mass with an irregular, lobulated surface and hyperintensity relative to the bladder muscle. (**C**, **D**) Post-gadolinium contrast images show strong, heterogeneous enhancement of the lesion with mild infiltration of the perirectal fat layer. REC, rectum; PR, prostate; BL, bladder; T1W, T1-weighted; T2W, T2-weighted; T1W FS, T1-weighted fat-saturated; Gd, gadolinium.

A 66-year-old male presented with a medical history of hepatitis B five years prior, chronic obstructive pulmonary disease diagnosed two years earlier, and an epididymal cyst ten years ago. Two months before admission, he experienced loose stools with mucus and blood occurring 2–3 times daily, accompanied by a 5-kg weight loss. Urinary function remained normal. Digital rectal examination revealed blood on the examining glove and a palpable mass approximately 10 cm from the anal verge, with a rough surface occupying approximately half of the rectal circumference. Colonoscopy identified a protruding mass in the middle third of the rectum, characterized by an irregular, ulcerated, and bleeding surface. Five tissue samples were collected for histopathological evaluation. Hematologic results were as follows: red blood cells, 4.81 T/L; hemoglobin, 135 g/L; white blood cells, 11.05 G/L; neutrophils, 7.38 G/L; and platelets, 270 G/L. Biochemical results showed aspartate aminotransferase (AST), 33.9 U/L; alanine aminotransferase (ALT), 24 U/L; alpha-fetoprotein (AFP), 8.9 IU/mL; and hepatitis B surface antigen (HBsAg), 2467 COI. Abdominal and cervical ultrasounds revealed no abnormalities. MRI was performed using a 1.5 Tesla scanner (SIGNA Creator, GE, USA) with the following pulse sequences: T2-weighted (T2W) PROPELLER (coronal, sagittal, and axial planes); T1-weighted (T1W) axial; DWI with b-values of 0 and 800 s/mm^2^; and T1-weighted fat-suppressed (T1W FS) coronal, sagittal, and axial sequences after intravenous administration of gadolinium-based contrast agent (Dotarem, Guerbet, France; 10 mL at 1 mL/s). Imaging demonstrated irregular thickening of the rectal wall approximately 10 cm from the anus over a 6-cm segment, with a rough, lobulated surface. The lesion appeared isointense on both T1W and T2W images. Post-contrast sequences showed strong but heterogeneous enhancement with invasion of the perirectal fat layer. No abnormal lymph nodes were observed (Fig. **1**). ADC maps were automatically generated using a monoexponential model with two b-values (0 and 800 s/mm^2^).

**Fig. (2):**
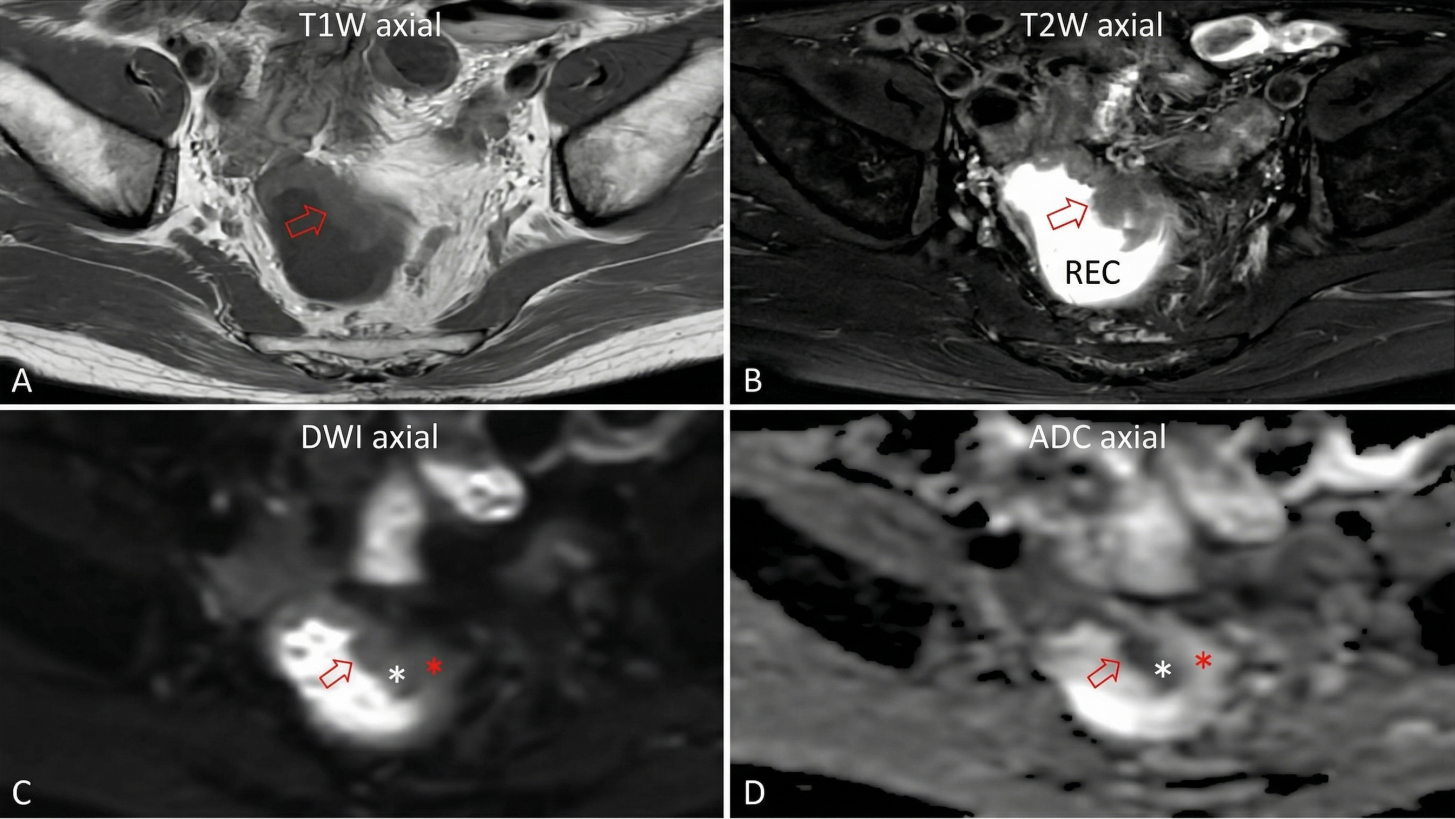
Axial pelvic MRI with different pulse sequences: (**A**, **B**) Axial T1W and T2W images show the tumor appearing isointense on both T1W and T2W images (red open arrow). (**C**, **D**) Diffusion MRI reveals two distinct tumor regions: (1) the surface demonstrates mild diffusion restriction, appearing slightly hyperintense on the DWI (gray) and hypointense on the ADC map (black) (white asterisk); and (2) the base shows no diffusion restriction, appearing hypointense on the DWI image (gray) and hyperintense on the ADC map (bright) (red asterisk). REC, rectum; T1W, T1-weighted; T2W, T2-weighted; DWI, diffusion-weighted imaging; ADC, apparent diffusion coefficient.

On diffusion MRI, the tumor surface appeared slightly hyperintense on the DWI and hypointense on the ADC map. In contrast, the tumor base did not exhibit hyperintensity on DWI or hypointensity on the ADC map, indicating the absence of diffusion restriction in this region (Fig. **2**). Circular regions of interest (ROIs) (≥ 2 mm^2^) were manually placed in both the diffusion-restricted (surface) and non-restricted (base) regions of the tumor to calculate mean ADC values. The measured mean ADC values were as follows: ROI 1 (surface), 0.82 × 10^-3^ mm^2^/s; and ROI 2 (base), 1.36 × 10^-3^ mm^2^/s. Despite the lack of diffusion restriction at the tumor base, the radiologists maintained a strong suspicion of malignancy.

**Fig. (3):**
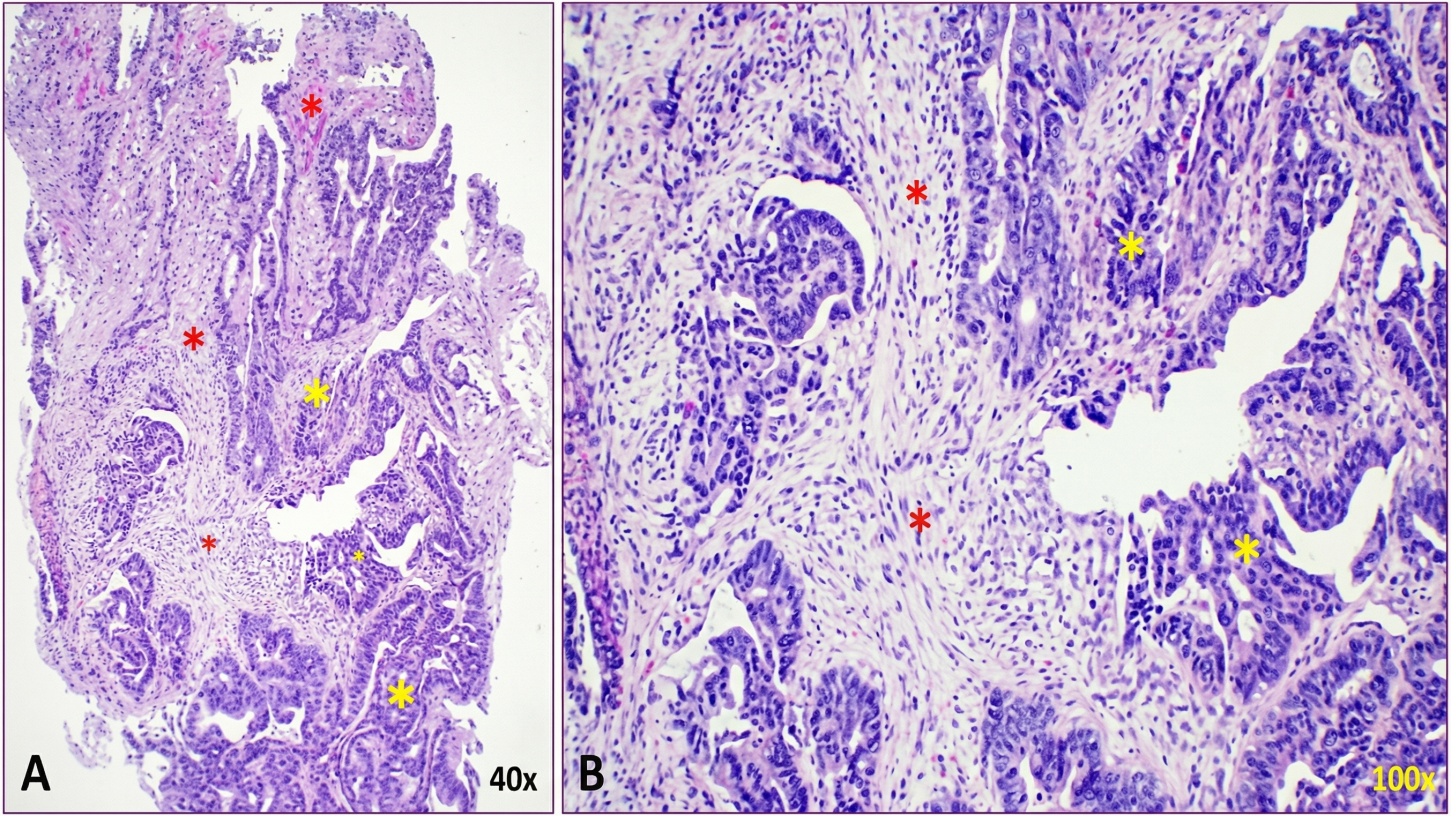
Histopathological images of rectal tumor stained with hematoxylin and eosin (H&E): (**A**) At 40× magnification, the tumor tissue exhibits architectural variability with numerous malformed glands, increased glandular density, irregular glandular arrangement (yellow asterisks), and stromal invasion (red asterisks). (**B**) At 100× magnification, cancerous glands display complex branching, papillary, or cribriform structures infiltrating the stroma. Tumor cell nuclei are large, pleomorphic, basophilic, and depolarized, with nuclei positioned toward the apical pole of the cells. The fibrous stroma is hyperplastic and infiltrated by chronic inflammatory cells. Pathological diagnosis: Moderately differentiated adenocarcinoma.

On histopathological examination with hematoxylin and eosin (H&E) staining, the tumor tissue consisted of cells with large, basophilic nuclei and an increased nuclear-to-cytoplasmic ratio. The cells were arranged in distorted glandular formations resembling a cribriform pattern. The fibrous stroma showed hyperplasia with infiltration of chronic inflammatory cells. The final diagnosis was moderately differentiated adenocarcinoma of the middle third of the rectum (Fig. **3**).

The patient underwent laparoscopic colon resection using a straight stapler. During surgery, the proximal resection margin was made approximately 12 cm from the tumor, and the distal margin was 6 cm away. The resected colon specimen was sent for histopathological analysis to reconfirm the tumor’s nature, evaluate malignancy grade, determine the extent of invasion, and assess margin status. Pathological findings were consistent with the preoperative diagnosis, confirming a moderately differentiated adenocarcinoma with perirectal fat infiltration. Both proximal and distal resection margins were free of tumor involvement.

## 3 DISCUSSION

Colorectal adenocarcinoma is a prevalent malignancy originating from the glandular epithelial cells of the colonic mucosa. Histologically, it is classified into five main types: (1) well-differentiated adenocarcinoma-composed of cells closely resembling normal tissue and demonstrating slow progression; (2) moderately differentiated adenocarcinoma-representing an intermediate form between well-differentiated and poorly differentiated types; (3) poorly differentiated adenocarcinoma-characterized by undifferentiated or poorly differentiated cells with high malignancy; (4) mucinous adenocarcinoma-containing abundant extracellular mucin, accounting for approximately 10–15% of cases, and associated with poor prognosis; and (5) signet-ring cell carcinoma-a rare, highly aggressive subtype with an extremely poor prognosis [6].

**Fig. (4):**
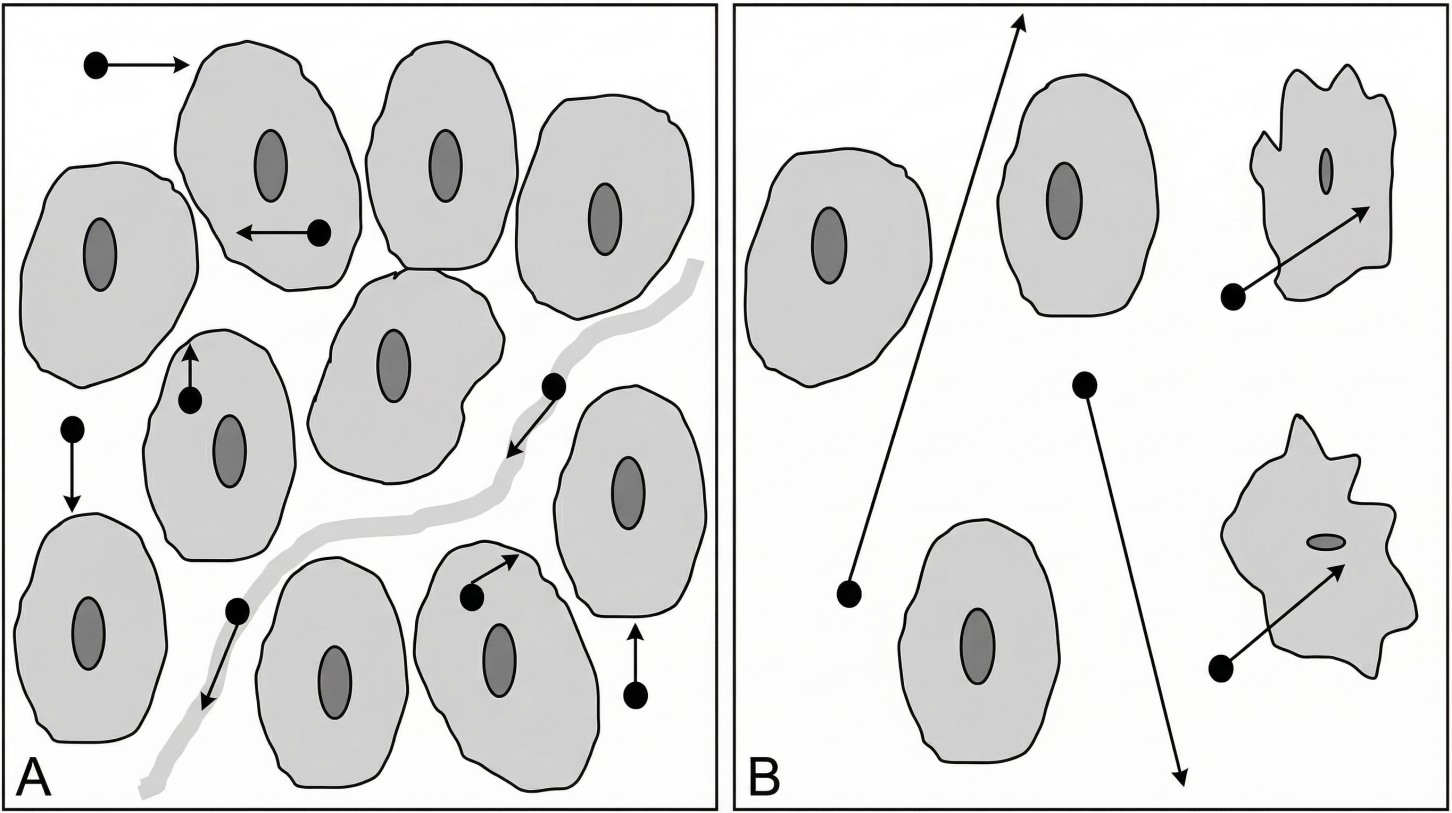
Diffusion of water molecules [8]. (**A**) Restricted diffusion: high cellularity and intact cell membranes. The illustration depicts one voxel of tissue assessed by DWI, containing cells and blood vessels. Water molecules (black circles with arrows) within extracellular, intracellular, and intravascular spaces contribute to the overall MR signal. In this densely cellular environment, water diffusion is limited due to reduced extracellular space and the presence of intact cell membranes that act as barriers to movement. (**B**) Free diffusion: low cellularity and disrupted cell membranes. In a less cellular environment, the relative expansion of extracellular space permits freer water diffusion. Damaged or permeable cell membranes further facilitate the exchange of water molecules between extracellular and intracellular spaces.

MRI serves as a primary diagnostic modality in evaluating rectal cancer due to the soft tissue composition of the rectum and the relative stability of pelvic organs. MRI provides high spatial resolution and excellent tissue contrast, enabling precise assessment of tumor location, morphology, size, depth of invasion, regional lymph node involvement, and distant metastases. DWI, ADC mapping, and contrast-enhanced sequences are particularly valuable in assessing tumor malignancy. In normal tissue, water molecules diffuse freely within the extracellular space. However, in malignant tissue, uncontrolled cellular proliferation increases cell density, narrows intercellular spaces, and restricts water molecule movement, leading to diffusion limitation or “water stagnation” (Fig. **4**). This restriction manifests as hyperintensity on DWI and hypointensity on ADC maps, serving as key imaging indicators for malignancy [7].

However, certain subtypes of colorectal adenocarcinoma, particularly mucinous and well-differentiated forms, may not exhibit restricted diffusion. This can be attributed to relatively low cell density or the presence of abundant mucus, which increases the extracellular space and facilitates freer water molecule movement. Consequently, these tumors often lack diffusion restriction on MRI [9-11].

The ADC quantifies the rate of water diffusion within tissue and is expressed in square millimeters per second (mm^2^/s). A low ADC value indicates restricted diffusion, typically resulting from high cell density (as seen in cancer) or enlarged cells due to edema (as in stroke). Conversely, a high ADC value reflects free diffusion, characteristic of normal tissues or lesions rich in fluid or mucus [12]. ADC values are derived by comparing signal intensities across at least two diffusion-sensitizing gradients (b-values); in this case, b = 0 and b = 800 s/mm^2^.

In the present case, the tumor exhibited a heterogeneous structure comprising two distinct regions: (1) the surface region demonstrated mild hyperintensity on the DWI and hypointensity on the ADC map, indicating higher cell density and more active cellular proliferation; and (2) the base region lacked hyperintensity on DWI and hypointensity on the ADC map, suggesting minimal diffusion restriction and relatively lower cell density. Histopathological examination corroborated these imaging findings, revealing areas with densely packed glandular structures and irregular arrangements adjacent to regions of looser stromal tissue. The boundary between these two regions was relatively well defined (Fig. **3**).

Clinically, some malignant tumors show uneven growth patterns. In well-vascularized regions, cells proliferate vigorously, increasing cellular density and producing strong diffusion restriction (hyperintense on DWI, hypointense on ADC). In contrast, poorly perfused regions-associated with relative hypoxia, necrosis, or stromal proliferation-display lower cell density, allowing freer water diffusion. Central necrosis or tissue degeneration may further reduce cellular density, facilitating water movement within the intercellular space, which decreases DWI signal intensity and increases ADC values. Although fibrotic stroma lacks high cellular density, its collagenous structure may partially impede water diffusion, resulting in pseudo-diffusion restriction and signal variation on DWI and ADC images [13]. The heterogeneous signal of cancerous tissue on diffusion MRI reflects underlying histologic heterogeneity within the tumor mass. This complexity complicates radiologic differentiation between benign and malignant lesions and underscores the need for close correlation between imaging and histopathologic findings. Biopsies often sample only limited tumor areas, which may not represent the full spectrum of malignancy. In contrast, MRI allows comprehensive, noninvasive assessment of the entire lesion, providing valuable information on regional tumor behavior. The integration of imaging and histopathologic analyses enhances diagnostic accuracy and aids in selecting appropriate therapeutic strategies [7].

Recent developments in digital medicine have significantly advanced colorectal cancer (CRC) management across diagnostic, perioperative, and follow-up stages. Deep learning models, particularly convolutional neural networks, have achieved expert-level performance in histopathologic and imaging interpretation, enabling automated lesion detection and molecular profiling [14]. Biomarkers such as butyrylcholinesterase show potential in predicting postoperative complications, improving risk stratification and individualized treatment planning [15]. Furthermore, integration of the Internet of Things supports real-time patient monitoring, robotic-assisted surgery, and telemedicine-based follow-up, enabling early detection of complications and sustained long-term surveillance. Collectively, these technologies mark a transition toward intelligent, data-driven, and patient-centered CRC care [16].

## CONCLUSION

This case demonstrates that moderately differentiated rectal adenocarcinoma may exhibit heterogeneous diffusion restriction and variable cellular density. Quantitative ADC analysis offers objective assessment of regional differences, reducing the risk of false-negative interpretation in areas with lower cellularity. Multiparametric MRI, incorporating T2-weighted and contrast-enhanced sequences, remains vital for accurate diagnosis, comprehensive tumor characterization, and effective treatment planning.

## Data Availability

The data and supportive information are available within the article.

## LIST OF ABBREVIATIONS

REC: Rectum
PR: Prostate
BL: Bladder
T1W: T1 Weighted
T2W: T2 Weighted
T1W FS: T1 Weighted Fat-Saturated
Gd: Gadolinium
DWI: Diffusion-Weighted Imaging
ADC: Apparent Diffusion Coefficient

## STANDARDS OF REPORTING

CARE guidelines were followed.
